# Towards Understanding the Effective Use of Antibiotics for Sepsis

**DOI:** 10.1016/j.chest.2021.04.038

**Published:** 2021-04-24

**Authors:** Michiel Schinkel, Ketan Paranjape, Justin Kundert, Rishi S. Nannan Panday, Nadia Alam, Prabath W.B. Nanayakkara

**Affiliations:** a Section of General and Acute Internal Medicine, Department of Internal Medicine, Amsterdam Public Health Research Institute, Amsterdam UMC, VU University Medical Center, Amsterdam, The Netherlands; b Center of Experimental and Molecular Medicine (C.E.M.M.), Amsterdam UMC, location Academic Medical Center, Amsterdam, The Netherlands; c Roche Diagnostics Corporation, Indianapolis, IN; d Center for Population Health Sciences, Lee Kong Chian School of Medicine, Nanyang Technological University, Singapore

**Keywords:** age, antibiotics, machine learning, mortality, PHANTASi trial, prehospital, sepsis, ARI, Adjusted Rand, CH, Calinski-Harabasz, PHANTASi, Prehospital Antibiotics Against Sepsis

## Abstract

**Background:**

The benefits of early antibiotics for sepsis have recently been questioned. Evidence for this mainly comes from observational studies. The only randomized trial on this subject, the Prehospital Antibiotics Against Sepsis (PHANTASi) trial, did not find significant mortality benefits from early antibiotics. That subgroups of patients benefit from this practice is still plausible, given the heterogeneous nature of sepsis.

**Research Question:**

Do subgroups of sepsis patients experience 28-day mortality benefits from early administration of antibiotics in a prehospital setting? And what key traits drive these benefits?

**Study Design and Methods:**

We used machine learning to conduct exploratory partitioning cluster analysis to identify possible subgroups of sepsis patients who may benefit from early antibiotics. We further tested the influence of several traits within these subgroups, using a logistic regression model.

**Results:**

We found a significant interaction between age and benefits of early antibiotics (*P* = .03). When we adjusted for this interaction and several other confounders, there was a significant benefit of early antibiotic treatment (OR, 0.07; 95% CI, 0.01-0.79; *P* = .03).

**Interpretation:**

An interaction between age and benefits of early antibiotics for sepsis has not been reported before. When validated, it can have major implications for clinical practice. This new insight into benefits of early antibiotic treatment for younger sepsis patients may enable more effective care.

**Take-home Points**

**Study Question:** Are there specific subgroups of patients with sepsis who are more likely to benefit from early antibiotic treatment?

**Results:** We found a significant interaction between age and benefits of early antibiotics, associating early treatment with a significant decrease in 28-day mortality among younger patients with sepsis.

**Interpretation:** Our results suggest that we should immediately consider antibiotic treatment in younger patients, whereas early treatment does not seem to have much beneficial effect in older sepsis patients.

Sepsis is a major health problem worldwide. A recent study estimated the global incidence of sepsis to be nearly 50 million cases annually, with 11 million sepsis-related deaths.^1^ Dysregulation of the host response to infections can cause organ dysfunction and subsequently leads to these high mortality rates.^2^ Sepsis is a truly heterogeneous syndrome,^3^^,^^4^ caused by different pathogens at various sites (eg, respiratory tract, urinary tract, or abdominal), which makes it difficult to develop general guidelines that will benefit all patients with sepsis.

Researchers have aimed to identify specific subgroups of sepsis patients to tailor the treatment. Seymour and colleagues,^5^ for example, categorized four clinical sepsis phenotypes with similar traits, that may also respond similarly to certain treatments.^5^ Current sepsis treatment mainly includes administration of antibiotics and IV fluids. The subcategorization of sepsis patients could help in using these options more effectively and giving to the right patient at the right time.

Most patients suspected of having systemic infections receive antibiotic treatment immediately in the ED. There is a long-standing belief that every hour of delay in administration of antibiotics leads to an increased risk of mortality, as suggested by Kumar et al^6^ in 2006. Many treatment protocols for sepsis have been guided by this belief, ultimately resulting in an international effort called the Surviving Sepsis Campaign guideline 1-hour bundle.^7^

Recently the benefits of early antibiotic treatment in all patients with suspected sepsis have been questioned.^8^, ^9^, ^10^, ^11^ Physicians are forced to sacrifice diagnostic accuracy to treat these patients early, which contributes to overuse of antibiotics.^8^^,^^12^^,^^13^ A Dutch study reported that 29% of suspected sepsis patients in the ED were unlikely to even have an infection.^12^ In a recent review, we evaluated the literature on the benefits of early antibiotics for sepsis and concluded that the evidence for this is mainly derived from observational studies.^8^ The only randomized controlled trial on this subject, called the Prehospital Antibiotics Against Sepsis (PHANTASi) trial, conducted by our research group, did not show significant benefits of early antibiotic treatment in a prehospital setting.^14^

Although no conclusive evidence supports the early use of antibiotics in all patients with suspected sepsis, subgroups of patients may benefit from early antibiotic treatment. In this study, we aim to identify subgroups of patients in the PHANTASi trial cohort who are likely to benefit from early antibiotic treatment and study their key traits, using machine learning.^15^

## Study Design and Methods

### Database

The PHANTASi trial database was used for this study.^14^ The PHANTASi trial randomized 2,672 patients with suspected sepsis to receive antibiotic treatment either in the ambulance (intervention) or once the patient had arrived in the ED (control). This resulted in a median difference in time to antibiotics of 96 minutes (interquartile range, 36-128) between the groups. The study ran between June 2014 and June 2016. Patients were included when they were at least 18 years of age, were suspected of having an infection, and had at least two Systemic Inflammatory Response Syndrome criteria, with a mandatory temperature ≥38 °C or ≤36 °C. The original trial was registered at ClinicalTrials.gov, number NCT01988428.^16^ More details on this study can be found here.^14^^,^^17^

Vital parameters and laboratory results were recorded in the ambulance and in the ED. Any treatments, including an early dose of antibiotics in the ambulance in the intervention group, were recorded. Diagnoses were confirmed by an expert panel, and sepsis severity was categorized according to the 2001 international sepsis criteria,^18^ which were the gold standard at the time. The study was powered to detect differences in the primary outcome, which was 28-day mortality.^14^

## Statistical Analysis

Statistical analyses were performed in R 3.5,^19^ and in R modules within the Alteryx software (Alteryx Inc),^20^ which is an extraction transformation and loading application. Differences between non-normally distributed and continuous variables were assessed with a Mann-Whitney *U* test.^21^ Differences between categorical variables were tested with a χ^2^ test. Normality of the data was assessed with histograms and Q-Q plots. A two-tailed *P* < .05 was considered statistically significant.

Machine learning algorithms were used to conduct exploratory partitioning cluster analysis to identify possible factors impacting the benefits of early antibiotic treatment. This clustering approach involved three broad phases: exploratory data analysis, preliminary cluster diagnostics, and then focused cluster partitioning based on key traits.

During the exploratory data analysis, unsupervised machine learning techniques (K-means, K-medians, and Neural Gas clustering) were performed to identify any relevant cluster patterns exhibited by combinations of traits with either known or suspected associations with 28-day mortality. Twenty-two exploratory analyses were performed involving various traits (outlined in e-Table 1: Exploratory K-Centroids Diagnostic Data Mining Trials). These clusters assessed various clinical factors obtained in the ambulance or ED, as well as deterioration between ambulance and ED (delta in particular traits such as heart rate, respiratory rate, and so forth). We visually assessed each cluster pattern outcome to gain general insight and help shape the direction of subsequent, more focused, clustering techniques.

We identified three specific focused clustering combinations, outlined in Table 1, for further evaluation and subsequent cluster diagnostics, based specifically on clinical factors obtained in the ambulance. A thorough pre-assessment K-Centroid diagnostic analysis was performed for these specific combinations of key traits. This involved identifying possible traits that could have a strong cluster relationship, and then algorithmically evaluating the mathematically ideal number of clusters (k) for each combination. Cluster diagnostic results, including supporting Adjusted Rand (ARI) and Calinski-Harabasz (CH) indexes for each selected k-value, are represented in Table 1. The ARI was used to help provide a measure of agreement, or similarity, between partitions; the CH provided a measure for separation and inter-cluster density. The assessment process evaluated the suitable number of clusters (k) by maximizing ARI and CH, when compared with k alternatives, to increase cluster performance and quality. Once the number of clusters was determined for each possible trait combination, the clustering assignment was attempted and associated to each patient record. We used K-Means clustering for each grouping, and no additional unit standardization was applied to input fields. See Table 1 for further details. These cluster analyses focused primarily on better understanding previously unknown relationships within the data, as well as to help focus the direction of subsequent, more traditional, multivariable logistic regression statistical analysis.

**Table 1:** K-Centroids Cluster Diagnostics

K-Centroids Method	Min/Max Cluster Parameter	No. of Traits Evaluated	Traits Assessed	No. of Clusters (k) for Partitioning	Diagnostic Results		Cluster	Cluster Results			
					Adjusted Rand (Mean)	Calinski-Harabasz (Mean)		Size	Average Distance	Max Distance	Separation
K-means	2/8	6	Heart rate (ambulance); systolic BP (ambulance); diastolic BP (ambulance); respiratory rate (ambulance); temperature (ambulance); blood oxygen saturation (ambulance)	3	0.61	342.11	1	1,290	99.28	2,691.1	34.4
							2	54	135.46	2,694.8	982.3
							3	1,175	53.94	1,016	33.3
K-means	2/8	2	Heart rate (ambulance); temperature (ambulance)	5	0.80	5,266.8	1	734	5.92	13.5	8.87
							2	865	3.34	8.02	7.66
							3	182	6.88	31.27	11.33
							4	130	10.31	58.13	14.49
							5	608	4.2	10.84	8.12
K-means	2/8	3	Age; heart rate (ambulance); temperature (ambulance)	2	0.93	4,485.1	1	1671	5.29	19.34	12.43
							2	848	8.59	39.58	11.66

To further test associations between 28-day mortality and various traits, a multivariable logistic regression model was used. The raw model was adjusted for confounders using the 10% change-in-estimate criterion, as is one of the accepted methods of confounder identification.^22^^,^^23^ Also, full models with all a priori identified theoretical confounders are presented.^24^

In some cases, age was used not as a continuous variable, but as a dichotomous variable. Categories were created by splitting the dataset in the 50% youngest and 50% oldest patients, to obtain equally large numbers of patients in both groups.^23^ The age ranges in these groups were 18 to 75 and 76 to 100 years, respectively.

## Results

### Exploratory Partitioning Cluster Analysis

**Figure 1:**
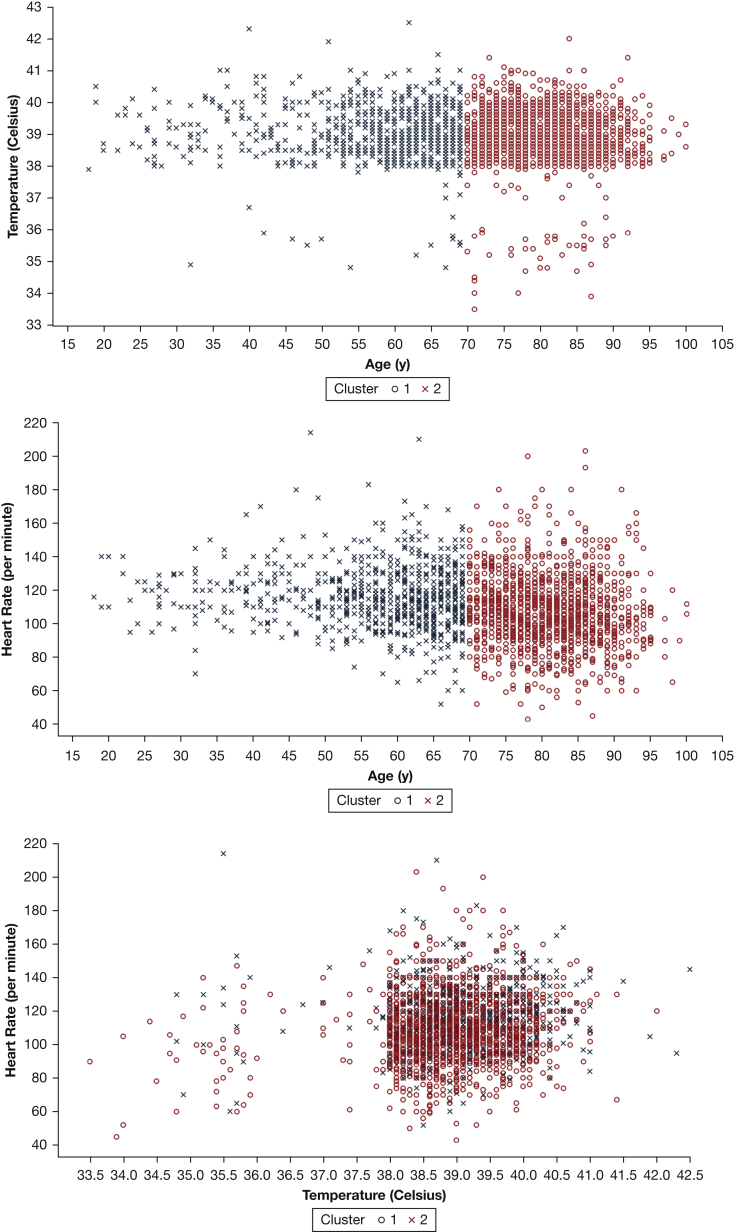
Three two-dimensional visualizations of the same clusters with k-means clustering based on age, heart rate, and temperature.

Clusters of similar patients were created based on various patient characteristics and with the use of various unsupervised machine learning techniques. Based on the most favorable Rand index values, a K-means cluster algorithm based on age, heart rate in the ambulance, and temperature in the ambulance was selected to generate two clusters (mean ARI, 0.93; mean CH, 4,485.1). The patterns produced using this model consistently resulted in strong ties associated with the age trait, seen in Figure 1, with partitioning occurring around the age of 70 years. Figure 1 illustrates three different two-dimensional representations of the same clusters, generated based on age, heart rate, and temperature. Although these are simplified representations of the three-dimensional clusters, they clearly show that the age trait is the most important driver of the clusters.

**Figure 2:**
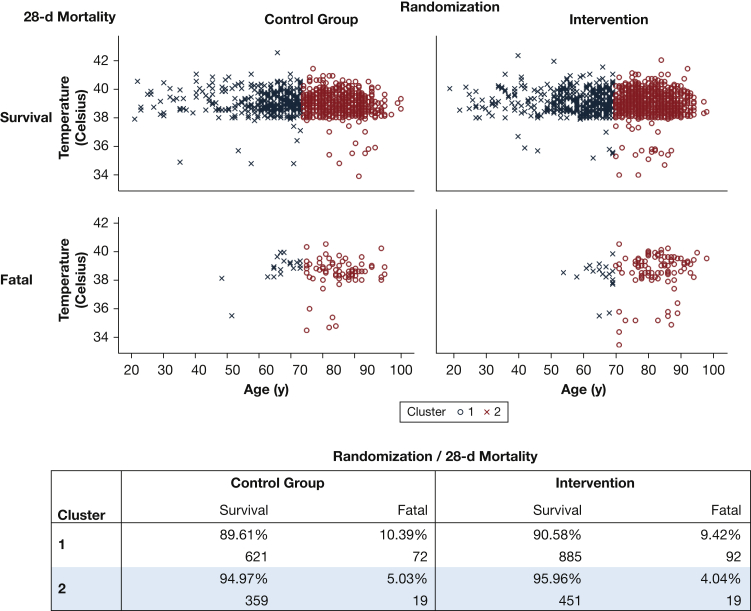
A, Visualization of clusters with k-means clustering based on age and heart rate (with temperature as the third clustering variable) segmented by intervention status and mortality outcome. B, Mortality rate summary percentages with k-means clustering based on age, heart rate, and temperature segmented by intervention status.

In Figure 2A, patients were categorized based on designated cluster and separated by randomization group and 28-day mortality outcome. For simplicity, we opted to only present a two-dimensional representation in this figure, because further insights are mostly derived from the age axis. The figure identifies the control group (antibiotics administered in the ED) from the intervention group (antibiotics in the ambulance) and separates patients who survived after 28 days from those deceased. Cluster 1 (denoted: O) resulted in 1,671 patients with a mean age of 80.6 years. Cluster 2 (denoted: X) produced 848 patients with a mean age of 57.5 years. One hundred fifty-three patients were categorized as outliers based on inconclusive clinical factors, and they were not assigned a cluster. Additional analysis shows that younger patients seen in cluster 2 may exhibit a slight lowering of the overall 28-day mortality rate in the intervention group (4.0%) when compared with younger patients in the control group (5.0%), whereas this is less pronounced in cluster 1 with older patients. Mortality rate percentages associated with each cluster are further outlined in Figure 2B.

### Logistic Regression Modeling

We created an association model to quantify the initial finding of a possible interaction between age and the effect of early antibiotic treatment. We used a logistic regression model to explain 28-day mortality in all patients who were categorized as having sepsis (n = 2,617). This number differs from the complete population (n = 2,672), because some patients had diagnoses other than sepsis in retrospect. Baseline characteristics of the included patients are presented in Table 2.

**Table 2:** Baseline Characteristics of the Complete Sepsis Population

Characteristic	Control Subject (n = 1,113)	Intervention (n = 1,504)	Total (N = 2,617)	*P*
Age, y				.509
Median (IQR)	75.0 (65.0, 83.0)	76.0 (66.0, 83.0)	76.0 (65.0, 83.0)	
Sex				.763
Male	638 (57%)	871 (58%)	1,509 (58%)	
Female	475 (43%)	633 (42%)	1,108 (42%)	
Youngest or oldest half of the patients				.536
Younger than 76 y	559 (50%)	737 (49%)	1,296 (50%)	
76 y or older	554 (50%)	767 (51%)	1,321 (50%)	
Sepsis severity				.341
Nonsevere sepsis	424 (38%)	576 (38%)	1,000 (38%)	
Severe sepsis	653 (59%)	863 (57%)	1,516 (58%)	
Septic shock	36 (3%)	65 (4%)	101 (4%)	
Charslon Comorbidity Index				.988
Median (IQR)	1.0 (1.0, 3.0)	1.0 (0.0, 3.0)	1.0 (1.0, 3.0)	
Do not resuscitate order				.307
No	666 (61%)	862 (59%)	1,528 (60%)	
Yes	425 (39%)	598 (41%)	1,023 (40%)	
Quick Sequential Organ Failure Assessment Score (qSOFA)				.003
≥2	176 (17%)	310 (22%)	486 (20%)	
<2	855 (83%)	1,109 (78%)	1,964 (80%)	
Use of immunosuppressive medication				.799
No	960 (86%)	1,292 (86%)	2,252 (86%)	
Yes	153 (14%)	212 (14%)	365 (14%)	
Patient already on oral antibiotics before randomization				.241
No	864 (79%)	1,189 (81%)	2,053 (80%)	
Yes	224 (21%)	274 (19%)	498 (20%)	
Pathogen resistant to ceftriaxone				.015
Sensitive	1,106 (100%)	1,483 (99%)	2,589 (100%)	
Resistant	0 (0%)	8 (1%)	8 (0%)	
Blood culture results from ambulance/ED				< .001
Negative	829 (75%)	1,239 (83%)	2,068 (80%)	
Positive	277 (25%)	252 (17%)	529 (20%)	
28-d mortality				.753
Survived	1,021 (92%)	1,386 (92%)	2,407 (92%)	
Died	91 (8%)	118 (8%)	209 (8%)	

IQR = interquartile range.

We used 28-day mortality as a dependent variable and intervention with early antibiotics (yes/no) as the main independent variable in our model. We also added the interaction between intervention and age (as a continuous variable) in the raw model, because this was the effect modifier we aimed to study. In the raw model, the effect of the intervention on 28-day mortality (OR, 0.13; 95% CI = 0.02-1.10; *P* = .061) as well as the interaction term between age and the benefit of the intervention (OR, 1.03; 95% CI = 1.00-1.05; *P* = .066) did not meet traditional measures of clinical significance. We then adjusted the model for a priori selected potential confounders, based on the 10% change-in-estimate criterion. This resulted in an adjustment based on Quick Sequential Organ Failure Assessment Score and Charlson comorbidity index, after which other variables did not meaningfully change this adjusted model. The adjusted model showed a significant benefit of the intervention on 28-day mortality (OR, 0.07; 95% CI, 0.01-0.79; *P* = .03) as well as a significant interaction term between age and the benefit of the intervention (OR, 1.03; 95% CI, 1.00-1.06; *P* = .03). Additionally, we created a full model based on all a priori selected potential confounders, irrespective of their influence in this dataset. This approach has been proposed in the literature and provided similar results as the adjusted model, as can be seen in Table 3, which also shows the full list of variables that we had selected as possible confounders.

**Table 3:** Associations of Various Traits With 28-Day Mortality Through Logistic Regression Modeling

Characteristics	Age Continuous						Age Dichotomous					
	Raw Model		Adjusted Model		Full Model		Raw		Adjusted Model		Full Model	
	OR (95% CI)	*P*	OR (95% CI)	*P*	OR (95% CI)	*P*	OR (95% CI)	*P*	OR (95% CI)	*P*	OR (95% CI)	*P*
Intervention (Y)	0.13 (0.02-1.10)	.061	0.07 (0.01-0.79)	.031	0.07 (0.01-0.80)	.031	0.68 (0.02-1.10)	.126	0.63 (0.36-1.06)	.082	0.59 (0.34-1.03)	.063
Age	1.03 (1.01-1.05)	.001	1.03 (1.01-1.05)	.008	1.00 (0.99-1.03)	.583	1.77 (1.14-2.77)	.012	1.60 (1.00-2.59)	.053	0.90 (0.54-1.51)	.679
Age∗intervention	1.03 (1.00-1.05)	.066	1.03 (1.00-1.06)	.033	1.03 (1.00-1.07)	.030	1.65 (0.90-3.05)	.110	1.89 (0.99-3.63)	.055	2.17 (1.11-4.30)	.025
Sex (female)					0.91 (0.66-1.24)	.543					0.92 (0.67-1.26)	.613
Charlson comorbidity index (per point increase)			1.17 (1.09-1.25)	.001	1.12 (1.04-1.20)	.002			1.18 (1.10-1.26)	<.001	1.12 (1.04-1.20)	.003
qSOFA (lower than 2)			0.46 (0.33-0.63)	.001	0.56 (0.40-0.78)	<.001			0.45 (0.33-0.62)	<.001	0.55 (0.39-0.77)	<.001
Do not resuscitate order (Y)					3.75 (2.58-5.55)	<.001					4.17 (2.88-6.14)	<.001
Antibiotics prior to hospital visit (Y)					1.34 (0.93-1.91)	.111					1.32 (0.91-1.88)	.132
Immunosuppressive comedication (Y)					1.48 (1.00-2.16)	.046					1.46 (0.98-2.13)	.056
Positive blood culture (Y)					1.37 (0.95-1.96)	.088					1.38 (0.95-1.97)	.084
Ceftriaxone resistant pathogen (Y)					2.83 (0.38-14.00)	.235					2.55 (0.33-13.35)	.230

qSOFA = Quick Sequential Organ Failure Assessment Score; Y = yes.

### Age as a Categorical Value

In the initial model, we used age as a continuous variable. Because we cannot be sure that the beneficial effects of early antibiotics decrease linearly with increasing age, we also created a model based on age groups. The age groups were created by a split based on the median age. This resulted in a cutoff at the age of 76. The raw model, with age as dichotomous variable, did not show significant benefits of the intervention (OR, 0.68; 95% CI, 0.02-1.10; *P* = .126), or interaction term between age and the benefit of the intervention (OR, 1.65; 95% CI, 0.90-3.05; *P* = .110). We then adjusted the model for the same variables as the adjusted model in the previous analysis, and we noticed differences in the benefits of early antibiotics (OR, 0.63; 95% CI, 0.36-1.06; *P* = .082), just as the interaction term between age and the benefit of the intervention (OR, 1.89; 95% CI, 0.99-3.63; *P* = .055) did not meet traditional measures of clinical significance. The full model, adjusted a priori with identified possible confounders, showed a similar benefit of early antibiotics as with age as a continuous variable (OR, 0.59; 95% CI, 0.34-1.05; *P* = .063) and the interaction term between age and the benefit of the intervention also presented similar results (OR, 2.17; 95% CI, 1.11-4.30; *P* = .025). See Table 3 for further details.

### Different Cutoff Values for Age Groups

In the analysis that used age as a dichotomous variable, we chose to split the groups based on the median age. e-Table 2 presents results for other cutoff values. Many cutoff values between 75 and 83 years of age showed significant results.

## Discussion

We reevaluated the PHANTASi trial cohort to identify subgroups of patients who may benefit from early antibiotic treatment and the traits driving these subgroups. We found a significant interaction between age and intervention with early antibiotics, associating early antibiotic treatment with a significant decrease in 28-day mortality among younger patients. We showed a significant interaction between age and the effect of early antibiotic treatment on mortality (*P* = .04). When we adjusted for this interaction, along with other potential confounders, a significant association was seen between intervention with early antibiotics and 28-day mortality (OR, 0.07; 95% CI, 0.007-0.75; *P* = .03).

### In Context

The three largest observational studies that evaluate the effect of time of antibiotic administration on mortality have not assessed the interaction between the age of the patients and the benefits of early antibiotic treatment.^25^, ^26^, ^27^ Over the past year, our research group has received several inquiries about the nonsignificant, but notably low, relative risk of mortality in the younger patients in the original PHANTASi trial, which spiked our interest in finding subgroups of patients who may have benefitted from early antibiotics. We opted to start this study by performing exploratory partitioning cluster analysis, rather than focusing specifically on age, because this allowed us to provide a broader view of potential patient factors that could be associated with benefits of early antibiotics treatment. However, we soon found that age seemed to be the most important driver of clusters and that we needed to focus on this trait.

### Residual Confounding

We tested the robustness of our results by using age as a continuous as well as a dichotomous variable, as well as using empirical and theoretical criteria to select the confounders we adjusted for. We thereby hoped to have limited residual confounding, which is inherent to secondary analyses. Because this study is based on secondary analyses, *P* values are difficult to interpret. The original study was not designed to detect this interaction, which makes it hard to find statistically significant results. We therefore focused on evaluating whether our findings remained similar when we examined different subgroups or adjusted the model for different potential confounders, while still providing *P* values and CIs for clarity.

We showed that the interaction between age and the intervention with early antibiotics was independent of the cutoff value we used for the age groups. In e-Table 2, we report *P* values for the interaction between age and intervention for cutoff levels between the ages of 70 and 85 years, which are significant at multiple thresholds. The absence of significant results at the lower and higher ends of that range is likely a reflection of the low numbers of patients and events in one of the two groups in those situations. This also can explain why the relative risk in the original publication of the PHANTASi trial did not reach statistical significance. The cutoff in the original publication was 65 years, which is a commonly accepted cutoff to define younger and older patients, but created a younger group (n = 600) that was considerably smaller than the elderly group (n = 2,017).

### Clinical Value

The interaction between age and benefits of early antibiotic treatment, which is associated with significant improvements in 28-day mortality in younger sepsis patients, can be clinically relevant. Knowing in which subcategory of patients benefits from early antibiotic treatment can be expected will enable effective and optimized care.

Our results suggest that we should immediately consider antibiotic treatment in younger patients, whereas early treatment does not seem to have much beneficial effects in older patients with sepsis. We do not propose a specific age cutoff for the benefits of early antibiotics, but we do believe that additional time to do a proper workup may be taken with elderly sepsis patients, to confirm the diagnosis before initiating antibiotic treatment. This is especially helpful because diagnosing sepsis in the elderly is often more challenging because of nonspecific presentations.^28^ Recent research indicates that early administration of antibiotics is associated with higher mortality when given to patients with greater diagnostic uncertainty.^29^ Arguably, the diagnostic uncertainty may be higher in elderly patients, given the nonspecific presentations. This provides an additional argument for withholding antibiotic treatment until the diagnosis is clearer.

We should note that our study only included patients with symptoms of sepsis. It may well be that early administration of antibiotics for elderly sepsis patients in practice is even less desirable, because this practice may even harm the patients with less specific presentations. Furthermore, there was only a small decrease in time to antibiotics (96 minutes) by intervening with antibiotics in the ambulance in this trial. In many settings, administration of antibiotics in the ambulance will result in larger decreases in time to antibiotics, which is possibly associated with an even stronger mortality benefit.

### Strengths

We examined an interaction that to our knowledge has never been reported before. The interaction between age and benefits of early antibiotic treatment may explain part of the variance in benefits of early antibiotic treatment that is observed throughout the literature on this subject.^3^^,^^30^ Furthermore, we used data from the single randomized trial on this subject, which lowers the chance of residual. Finally, we could evaluate the effect of potential confounders such as antibiotic sensitivities, whereas most studies on this subject lack these important data to evaluate adequacy of antibiotic treatments.^31^

### Limitations

We recognize the limitations of performing secondary analyses. Subgroup effects can be misleading and can be explained by chance.^32^ To minimize the risk that we found these results by chance, we performed several different analyses to see whether our results were robust. A second limitation is that we were not able to validate our findings in a similar cohort, because the PHANTASi trial was the only randomized trial on this subject and was conducted in a very specific setting. Validation of our findings in existing large observational cohorts could provide additional strength to our findings. However, such cohorts carry high risk of residual confounding and will not be able to undeniably validate or disprove our findings. A definite answer to whether young patients benefit from early antibiotics can only be given by another randomized study such as the PHANTASi trial.

## Interpretation

In conclusion, we have re-examined the effects of early antibiotic treatment for sepsis, finding a significant interaction between age and mortality benefits of this practice. Young patients with sepsis seem to experience a significant mortality benefit from early antibiotic treatment in the ambulance, which lessens as age increases. This interaction has not been reported before. Validation studies in other cohorts are needed to confirm our findings, which could lead to a shift in the way we think about the pathophysiology of sepsis and the most optimal treatment strategies.

## Supplementary Data

e-Online Data
