# Supplementary material for: Towards Understanding the Effective Use of Antibiotics for Sepsis
Source: Chest. 2021 Apr 24;160(4):1211–21. doi: 10.1016/j.chest.2021.04.038 (PMC8546240; doi:10.1016/j.chest.2021.04.038)
Supplement: e-Online Data [file mmc1.pdf]

CHEST Online Supplement

Towards Understanding the Effective Use of Antibiotics for Sepsis

Michiel Schinkel, MD; Ketan Paranjape, MSc; Justin Kundert, MSc;
Rishi S. Nannan Panday, PhD; Nadia Alam, PhD; and Prabath W.B. Nanayakkara, MD, PhD

CHEST 2021; 160(4):1211-1221

Online supplements are not copyedited prior to posting and the author(s) take full responsibility for the accuracy of all data.

© 2021 AMERICAN COLLEGE OF CHEST PHYSICIANS. Reproduction of this article is prohibited without written permission from the American College of Chest Physicians. See online for more details. **DOI:** 10.1016/j.chest.2021.04.038

e-Table 1. Exploratory K-Centroids Diagnostic Data Mining Trials

Analysis Trial Number	K-Centroids Method	Min/Max Cluster Parameters	Number of Traits Evaluated	Traits Assessed	Number of Clusters for Partitioning (Based on Preliminary Diagnostics Assessment)
1	K-means	2/8	6	Sex; Age; Heart Rate (Ambulance); Respiratory Rate (Ambulance); Temperature (Ambulance); Blood Oxygen Saturation (Ambulance)	4
2	K-medians	2/8	6	Heart Rate (Ambulance); Systolic BP (Ambulance); Diastolic BP (Ambulance); Respiratory Rate (Ambulance); Temperature (Ambulance); Blood Oxygen Saturation (Ambulance)	3
3	K-medians	2/8	2	Sex; Age	8
4	K-means	2/8	2	Heart Rate (Ambulance); Temperature (Ambulance)	5
5	K-means	2/8	2	Age; Temperature (Ambulance)	3
6	K-means	2/8	3	Age; Heart Rate (Ambulance); Temperature (Ambulance)	2
7	K-means	2/8	2	Age; Temperature (ED)	4
8	K-means	2/8	2	Heart Rate (ED); Temperature (ED)	3
9	K-means	2/8	6	Heart Rate (ED); Systolic BP (ED); Diastolic BP (ED); Respiratory Rate (ED); Temperature (ED); Blood Oxygen Saturation (ED)	2
10	Neural Gas	2/10	3	Age; Heart Rate (ED); Temperature (ED)	2
11	K-means	2/8	13	Sex; Age; Heart Rate (Ambulance); Systolic BP (Ambulance); Diastolic BP (Ambulance); Respiratory Rate (Ambulance); Temperature (Ambulance); Blood Oxygen Saturation (Ambulance); Delta Heart Rate (Ambulance > ED); Delta Systolic BP (Ambulance > ED); Delta	2

Online supplements are not copyedited prior to posting and the author(s) take full responsibility for the accuracy of all data.

				Diastolic BP (Ambulance > ED); Delta Respiratory Rate (Ambulance > ED); Delta Blood Oxygen Sat (Ambulance > ED)	
12	K-means	2/8	3	Age; Respiratory Rate (Ambulance); Delta Respiratory Rate (Ambulance > ED)	2
13	K-means	2/8	2	Heart Rate (Ambulance); Delta Heart Rate (Ambulance > ED)	
14	K-means	2/8	4	Respiratory Rate (Ambulance); Delta Respiratory Rate (Ambulance > ED); Heart Rate (Ambulance); Delta Heart Rate (Ambulance > ED)	2
15	K-medians	2/8	4	Respiratory Rate (Ambulance); Delta Respiratory Rate (Ambulance > ED); Heart Rate (Ambulance); Delta Heart Rate (Ambulance > ED)	2
16	K-means	2/8	3	C-reactive Protein (ED Lab); Leucocytes (ED Lab); Creatinine (ED Lab)	4
17	K-means	2/8	2	Age; Temperature (ED)	3
18	K-means	2/8	3	Delta Heart Rate (Ambulance > ED); Delta Respiratory Rate (Ambulance > ED); Delta Blood Oxygen Sat (Ambulance > ED)	2
19	K-means	2/8	2	Heart Rate (ED); Temperature (ED)	2
20	K-means	2/8	5	Age; Heart Rate (Ambulance); C-reactive Protein (ED Lab); Leucocytes (ED Lab); Creatinine (ED Lab)	4
21	K-means	2/8	5	Age; Heart Rate (Ambulance); C-reactive Protein (ED Lab); Leucocytes (ED Lab); Creatinine (ED Lab)	4
22	K-means	2/8	3	C-reactive Protein (ED Lab); Leucocytes (ED Lab); Creatinine (ED Lab)	4

Online supplements are not copyedited prior to posting and the author(s) take full responsibility for the accuracy of all data.

e-Table 2. P-values of the interaction term between age and intervention for different cut-off values for age in the full model

Cut-off (years)	P-value interaction term	Odds ratio interaction term	Confidence interval	Number of young patients	Number of elderly patients
70	0.255	1.58	0.72-3.47	887	1730
71	0.315	1.49	0.68-3.25	936	1681
72	0.222	1.57	0.76-3.27	992	1625
73	0.166	1.65	0.81-3.38	1073	1544
74	0.130	1.72	0.85-3.47	1132	1485
75	0.057	1.96	0.98-3.94	1202	1415
76	0.025	2.17	1.11-4.30	1296	1321
77	0.016	2.24	1.17-4.34	1388	1229
78	0.054	1.88	0.99-3.60	1481	1136
79	0.111	1.67	0.89-3.17	1583	1034
80	0.060	1.84	0.98-3.47	1666	951
81	0.171	1.56	0.93-2.94	1767	850
82	0.035	2.00	1.05-3.83	1852	765
83	0.041	1.98	1.03-3.83	1932	685
84	0.205	1.54	0.79-3.02	2015	602
85	0.135	1.71	0.85-3.49	2110	507

Online supplements are not copyedited prior to posting and the author(s) take full responsibility for the accuracy of all data.
